# Exploring hidden diversity in Southeast Asia’s *Dermogenys* spp. (Beloniformes: Zenarchopteridae) through DNA barcoding

**DOI:** 10.1038/s41598-018-29049-7

**Published:** 2018-07-17

**Authors:** Samsudin Nurul Farhana, Zainal Abidin Muchlisin, Thuy Yen Duong, Suwat Tanyaros, Larry M. Page, Yahui Zhao, Eleanor A. S. Adamson, Md. Zain Khaironizam, Mark de Bruyn, Mohd Nor Siti Azizah

**Affiliations:** 1 School of Biological Sciences, Universiti Sains Malaysia, 11800 Minden, Penang Malaysia; 2 Faculty of Marine and Fisheries, Syiah Kuala University, Banda Aceh, 23111 Indonesia; 3 College of Aquaculture and Fisheries, Cantho University, Cantho City, Vietnam; 4 Faculty of Science and Fisheries Technology, Rajamangala University of Technology Srivijaya, Trang campus,, 92150 Trang, Thailand; 5 Florida Museum of Natural History, Gainesville, 32611 Florida USA; 6 Institute of Zoology, Chinese Academy of Sciences, Datun Road, Chaoyang District, Beijing, 100101 People’s Republic of China; 7 The Natural History Museum, Cromwell Road, London, SW7 5BD United Kingdom; 8 The Fishmongers’ Company, Fishmongers’ Hall, London Bridge, London, EC4R 9EL United Kingdom; 9 The University of Sydney, School of Life and Environmental Sciences, Sydney, New South Wales Australia; 10 Institute of Marine Biotechnology, Universiti Malaysia Terengganu, 21030 Kuala Terengganu, Terengganu Malaysia

## Abstract

Members of the freshwater halfbeak genus *Dermogenys* are hard to identify to the species level, despite several previous attempts to isolate fixed meristic, morphometric and colour pattern differences. This has led to ongoing confusion in scientific literature, records of species occurrence, and entries in museum collections. Here, a DNA barcoding study was conducted on the genus to gain further understanding of its taxonomic status across the Southeast Asian region. Fish were collected from 33 localities, spanning freshwater and brackish habitats in Malaysia, Western Indonesia, Thailand and Vietnam. In total, 290 samples of *Dermogenys* spp. were amplified for a 651 base pair fragment of the mitochondrial cytochrome oxidase *c* subunit I (COI) gene. Analysis was able to successfully differentiate the three species: *D*. *collettei*, *D*. *siamensis*, *D*. *sumatrana*; reveal the presence of a new putative species, *Dermogenys* sp., that was sampled in sympatry with *D*. *collettei* at three locations; as well as uncovering two genetic lineages of a fifth species, *D*. *bispina*, that display non-overlapping geographical distributions in drainages of northern Borneo; Kudat and Sandakan. This study expands the barcode library for Zenarchopteridae, demonstrates the efficacy of DNA barcoding techniques for differentiating *Dermogenys* species, and the potential thereof in species discovery.

## Introduction

The tropical halfbeak genus *Dermogenys*, commonly known as silver halfbeak, pygmy halfbeak, wrestling halfbeak, Malayan halfbeak, or ‘julong julong’, occurs in the Southeast Asian region. The genus is the smallest in size among zenarchopterids, and inhabits fresh and brackish waters^1^, being able to tolerate some level of salinity. Halfbeaks are of limited economic value, however they are sometimes encountered in regional fish markets in Cambodia, Thailand and Vietnam.

As a group, *Dermogenys* has received considerable scientific attention^2–14^. The genus is viviparous (bearing live young) and generally limited to freshwater and estuarine habitats, suggesting low dispersal capacity; yet has a wide natural geographical distribution, including large mainland rivers such as the Mekong and drainages of the Indonesian and Philippine archipelagos. These factors mean the genus presents a tractable model for investigation of factors affecting phylogeographical pattern^12^ in what is a biogeographically complex and biologically diverse region of the tropics^15,16^.

Historically, *Dermogenys* taxonomy and systematics have been contentious and challenging. First described in the early 19^th^ Century, in van Hasselt^17^, the type specimen was referred to as *D*. *pusilla*. In the first revision, Mohr^18^ classified four of the then ten nominal species, considering three to be synonyms of *D*. *pusilla*. In a subsequent revision, Brembach^19^ recognized ten species and three subspecies, with diagnosis based on modified anal fin characters in males. He classified several populations that had consistent differences in the andropodium (male modified anal fin) as *D*. *pusilla*. This has led to erroneous classifications throughout museum collections and the literature^11^. Meisner & Collette^1^ named a new *Dermogenys* species from Sabah as *D*. *bispina*. This was followed by the discovery of a further four species, differentiated based on phylogenetic analysis of the gonadal histology and embryonic modifications associated with viviparity, and on osteological characters in modified anal fin rays^11^. Meisner concluded that the osteological characters of anal fin rays in males were diagnostic for species identification. Currently, there are 12 morphologically recognised species of *Dermogenys* in the Southeast Asian inland waters, as reviewed by Meisner^11^.

Among the halfbeaks, the genus level diagnostics as described by Rainboth^20^ are widely used as a guide for field identification. To aid identification at lower taxonomic rank, Meisner & Burns^9,10^ and Meisner^11^ developed keys for species level *Dermogenys* identification based on extensive morphological and morphometric analyses of a large number of anatomical structures. However, taxonomic differentiation among *Dermogenys* species remains challenging and is difficult in non-specialist facilities due to the small size of the individuals and overlapping morphological characteristics within the genus, requiring a considerable degree of skill and taxonomic expertise. For instance, Meisner^11^ concluded that the osteological characters are only informative at the generic level, while gonad histology yields characters useful for differentiating species groups within genera, as well as differentiating at genus level between *Dermogenys* and *Nomorhamphus*. According to Meisner^11^, the single diagnostic character for species level identification is the shape of the modified anal fin, however this character is only present in male specimens, and is only discernible through specialised radiography and optimal staining techniques. Although meristic and morphometric characters have proved efficient in resolving taxonomic questions at the species level in many teleost studies^21–30^, in the case of *Dermogenys*, they fail to adequately identify individuals to species level^11,19^. Likewise, characters like black pigmentation of pelvic and dorsal fins in males and melanophore arrangement from anterior to anal fin in females may aid in sorting the *Dermogenys* specimens into different groups at the genus level, but not to a precise taxon.

This study therefore attempts to alleviate these taxonomic challenges through a DNA barcoding approach. This technique, which relies on fixed differences in mitochondrial DNA sequences between taxa, has shown to be approximately 90% successful in species identification of freshwater and marine fishes^31–35^. Once a DNA barcoding database is established, the method offers a rapid way to allocate unknown specimens to correct species names, and can be used to identify individuals that lack defining morphological characters, for example females, larval fishes, or processed fish products^36–38^, among a range of other applications^39–42^. The ability for DNA barcoding to identify unknown specimens has proven useful across a wide range of biological disciplines, such as in biosecurity^43,44^, wildlife forensics^45–47^, phylogenetics^31,48–50^, and more generally in the conservation and management of wildlife^51–54^.

To date, the only *Dermogenys* barcode sequences available in online DNA databases and identified to species level are for *D*. *pusilla*, while all other *Dermogenys* barcodes come from fish that were only identified to genus level. Therefore, this study aims to generate new DNA barcodes for the species obtained during the course of this study, and to assign these to the species level using morphological and molecular tools, providing a robust taxonomic framework for future research on *Dermogenys*. The findings provide further insights into systematics as well as the phylogenetic relationships of the genus and highlight the complementarity of morphological and molecular characteristics in elucidating the taxonomic status and systematics of this group.

## Results

### Collection and morphological identification

**Figure 1:**
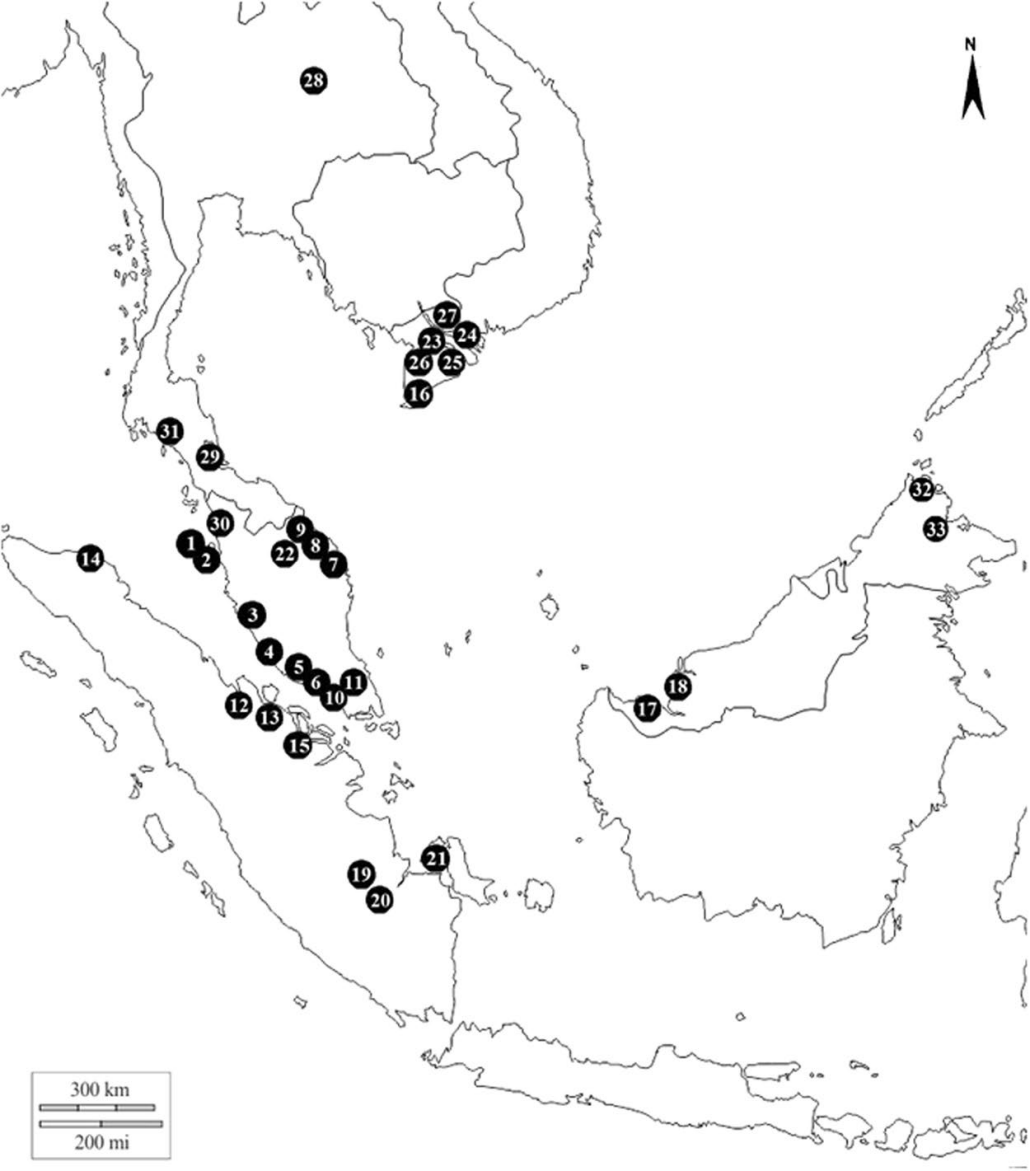
The localities of *Dermogenys* fish sampled from inland waters of Malaysia, Western Indonesia, Thailand and Vietnam. Refer to Table 1 for sampling details. Maps drawn and adapted by Adobe Photoshop CS3 from public domain image provided by D-maps.com (http://d-maps.com/m/asia/asiesudest/asiesudest06.svg).

Sample collection across 33 freshwater locations in Thailand, Vietnam, Malaysia and Western Indonesia yielded a total of 290 *Dermogenys* specimens (Fig. 1, Table 1). Island-wide sampling was conducted in Western Indonesia (Sumatra), however no *Dermogenys* specimen was obtained from the west coast of this island. Across all locations, most of the individuals collected (70%) were adults and therefore could be putatively assigned to their morphological species using the techniques outlined in the Methods section. This resulted in the identification of five morpho-species: *D*. *collettei* Meisner^11^ (30%), *D*. *sumatrana* Bleeker^55^ (14.8%), *D*. *siamensis* Fowler^56^ (17.6%), *D*. *bispina* Meisner & Collette^1^ (3.1%), and a newly observed group henceforth referred to as *Dermogenys* sp. (4.8%).

**Table 1:** *Dermogenys* samples used in this study. For map locations consult Fig. 1.

Map reference.	*Dermogenys* species	Locality name	Specimen code	n	K2P distance	Uncorrected p-distance
1	*D*. *collettei*	Kuala Sg. Pinang	KSP	10	0.000	0.000
2	*D*. *collettei*	Sg, Pulau Betong	KP	10	0.000	0.000
3	*D*. *collettei* (5) *Dermogenys* sp. (6)	Kuala Sepetang	SPP	11	**0.051**	**0.047**
4	*D*. *collettei*	Kuala Selangor	KSL	10	0.000	0.000
5	*D*. *collettei*	Sg. Kesang	SK	6	0.000	0.000
6	*D*. *collettei*	Sg. Muar	MR	11	0.000	0.000
7	*D*. *collettei*	Keluang	KET	3	0.000	0.000
8	*D*. *collettei*	Limbongan	LMT	11	0.002	0.002
9	*D*. *collettei*	Tok Bali	TBK	13	0.002	0.002
10	*D*. *collettei*	Simpang Ringgam*	DC652	1	n/c	n/c
11	*D*. *collettei*	Jemaluang*	DC683	1	n/c	n/c
12	*D*. *collettei* (4) *Dermogenys* sp. (1)	Sg. Iyu	SI	5	**0.036**	**0.034**
13	*D*. *collettei*	Sg. Siak	SIA	13	0.000	0.000
14	*D*. *collettei* (9) *Dermogenys* sp. (3)	Alur Itam	AI	12	**0.038**	**0.035**
15	*D*. *collettei*	Sg. Setayan	STY	10	0.000	0.000
16	*D*. *collettei*	Ca Mau	CM	5	0.001	0.001
17	*D*. *sumatrana*	Kuching	KUC	9	0.000	0.000
18	*D*. *sumatrana*	Igan	SS	10	0.004	0.004
19	*D*. *sumatrana*	Kebuk Karak	KK	23	0.000	0.000
20	*D*. *sumatrana*	Ogan	SO	2	0.000	0.000
21	*D*. *sumatrana*	Selidung Lama Gabak	SLD	11	0.003	0.003
22	*D*. *siamensis*	Sg. Nering	SN	10	0.000	0.000
23	*D*. *siamensis*	Can Tho	CT	6	0.013	0.013
24	*D*. *siamensis*	Travinh	TRV	7	0.000	0.000
25	*D*. *siamensis*	Soc Trang	ST	8	0.001	0.001
26	*D*. *siamensis*	Kiet Giang	KG	9	0.014	0.014
27	*D*. *siamensis*	Lang An	RP	10	0.001	0.001
28	*D*. *siamensis*	Nam Khem	XKT	14	0.003	0.003
29	*D*. *siamensis*	Patthalung	PAT	9	0.000	0.000
30	*Dermogenys* sp.	Sg. Lalang	SL	12	0.000	0.000
31	*Dermogenys* sp.	Sikao	RMU	6	0.002	0.002
32	*D*. *bispina* Kudat	Kudat	KDT	6	0.001	0.001
33	*D*. *bispina* Sandakan	Sandakan	SAN	6	0.002	0.002

Species names as verified using morphological and genetic information. Multiple species were present at three locations, as reflected by elevated genetic distances among individuals collected at these sites (indicated in bold).

^*^Sample obtained from Florida Museum of Natural History.

n/c – no calculation due to single sample.

Morphological identification among adults was not straightforward. Of the adults sampled, a combined total of 14 specimens from Sikao (Isthmus of Kra, Thailand), Alur Itam and Sungai Iyu (Western Indonesia), and Sungai Lalang and Kuala Sepetang (Peninsular Malaysia) could not be classified based on existing keys. At three of these sites (Alur Itam, Sungai Iyu and Kuala Sepetang), additional individuals were present that could be positively identified as *D*. *collettei*, so in this case co-occurring unidentified fishes were tentatively classified as being of the same species. In the other two instances (Sikao, *n* = 6, and Sungai Lalang, *n* = 12), no positive identifications could be made of any specimen, and therefore all were recorded as *Dermogenys* sp. No adult male was available from these locations, and although females were morphologically similar to *D*. *collettei*, they lacked the thin line of melanophores arranged from the anterior to anal fin characteristic of females of this species.

The remaining 86 (29.7%) samples were sub-adult specimens lacking morphological diagnostic characters, and as sub-adults are expected to co-exist with the adult specimens, where adults could be identified the identity of sub-adults was tentatively assumed to be the same. For all specimens, DNA barcodes were then employed to confirm (or challenge) morphological identifications.

### DNA Barcoding for species identification

All samples (290) were successfully DNA barcoded for a 651 bp segment of the mitochondrial COI gene. All sequences have been deposited in GenBank with accession number MG563383 – MG563672. There were no insertions/deletions or stop codons in the alignment, which had mean nucleotide frequencies of A = 23%, T = 31%, C = 27% and G = 19%, and 157 variable nucleotide positions, including 134 parsimoniously informative sites, with most variation occurring at third codon positions. A total of 53 different COI haplotypes were observed among all *Dermogenys* sequenced.

**Figure 2:**
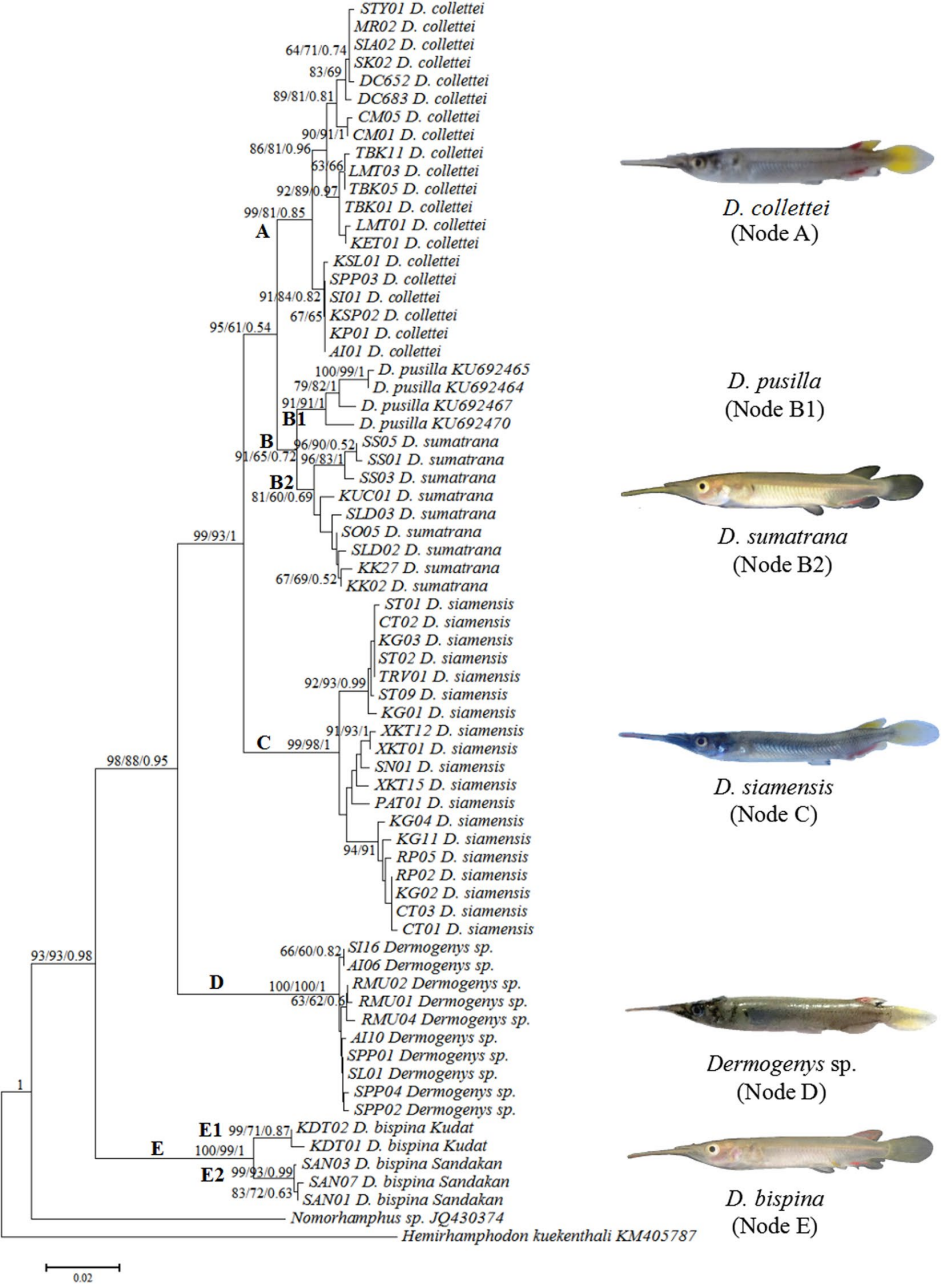
COI gene tree of six putative *Dermogenys* species. Values at nodes represents the bootstrap support and posterior probability (NJ/ML/BI). Gene tree includes sequences retrieved from GenBank for *D*. *pusilla* and for outgroups *Nomorhamphus* sp. (JQ430374) and *Hemirhamphodon kuekenthali* (KM405787) that were used to root the tree.

All sequences were identified as *Dermogenys* spp. by the GenBank BLAST tool, however, as prior to this study no species-level reference COI data existed for *Dermogenys* other than for *D*. *pusilla*, and as no *D*. *pusilla* was collected in this study, no individual could be identified to species level with the GenBank search. The current data set did, however, clearly exhibit variation consistent with five discrete monophyletic groups that all differed from *D*. *pusilla*, as visualised in Fig. 2. These groups were largely consistent with species designations based on morphology, identifying groupings for *D*. *collettei* (12 haploypes), *D*. *sumatrana* (12 haploypes), *D*. *siamensis* (15 haploypes), *D*. *bispina* (6 haploypes), and a newly observed taxon, *Dermogenys* sp. (8 haploypes).

Not all was as expected, however, with relatively high genetic distance observed within groups, tentatively identified samples showing identity to unexpected genetic clusters, and individuals collected at the same location showing identity to different groupings. This is reflected in within-site measures of Kimura 2-parameter (K2P) genetic distance, which ranged between 0.0% where all individuals had identical barcodes, to 5.1% where individuals collected together assigned to multiple genetic clusters, indicating more than one putative species was present in these sites (Table 1). Samples from three locations showed high “intrapopulation” K2P distance due to the presence of multiple putative species; Kuala Sepetang (5.1%), Sg. Iyu (3.6%) and Alur Itam (3.8%). Individuals from all three sites had previously been flagged as hard to identify based on morphology, and as a result of the barcode evidence, all specimens from each site were sorted into two groups; the *D*. *collettei* group and the *Dermogenys* sp. group (Table 1) and are considered discrete putative species in all further analysis and discussion.

Average K2P genetic distances among barcodes generated here, Genbank *D*. *pusilla* barcodes, and representatives of other zenarchopterids are presented in Table 2. Within putative taxa, as defined using a combination of morphological similarity and genetic monophyly, the lowest mean distance (0.02%) was observed for the unclassified *Dermogenys* sp. (*n* = 28), that was collected across five locations (Table 1, Fig. 1). Maximum average distance at the intraspecific level was 1.5% (for *D*. *bispina* and *D*. *pusilla*), while minimum average between species was 3.7% between *D*. *sumatrana* and *D*. *pusilla*.

**Table 2:** Interspecific and intraspecific mean genetic distances of K2P distance and p-distance (in parenthesis) for the six putative (based on morphology and COI) species of *Dermogenys* as identified by a combination of morphological and genetic data, and GenBank *D*. *pusilla*.

	Species	1	2	3	4	5	6	7	8
1	*D*. *collettei*	0.011 (0.011)							
2	*D*. *sumatrana*	0.038 (0.037)	0.008 (0.008)						
3	*D*. *siamensis*	0.063 (0.060)	0.061 (0.058)	0.014 (0.013)					
4	*Dermogenys* sp.	0.092 (0.085)	0.098 (0.090)	0.112 (0.102)	0.002 (0.002)				
5	*D*. *bispina*	0.110 (0.101)	0.120 (0.109)	0.126 (0.115)	0.127 (0.116)	0.015 (0.015)			
6	*D*. *pusilla*	0.045 (0.044)	0.037 (0.036)	0.071 (0.067)	0.096 (0.089)	0.120 (0.110)	0.015 (0.015)		
7	*Nomorhamphus* sp.	0.164 (0.138)	0.169 (0.142)	0.171 (0.148)	0.150 (0.134)	0.151 (0.136)	0.173 (0.144)	n/c (n/c)	
8	*H*. *kuekenthali*	0.218 (0.188)	0.235 (0.202)	0.214 (0.188)	0.212 (0.185)	0.198 (0.172)	0.232 (0.199)	0.202 (0.179)	n/c (n/c)

n/c – no calculation due to single sample.

**Figure 3:**
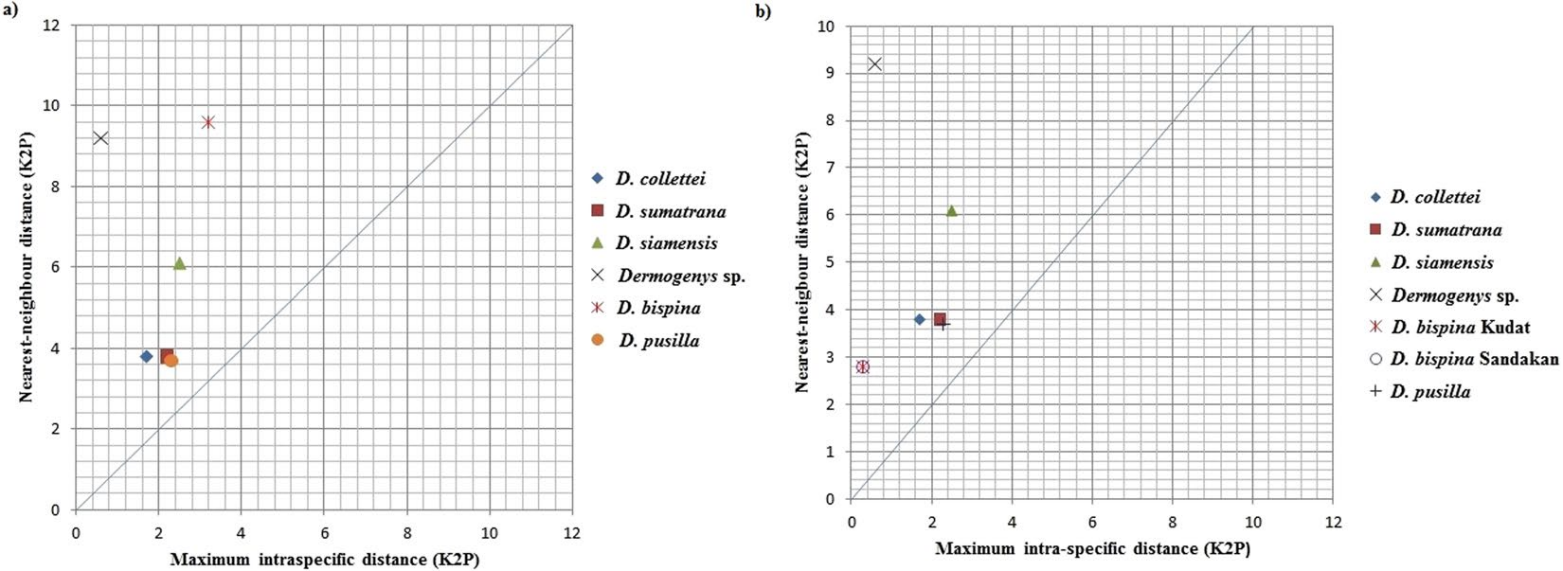
Maximum intraspecific distance (% K2P) plotted against nearest neighbour distance (% K2P) for the seven OTUs examined in this study. Points above the line indicate species with a barcode gap. (**a**) Six initial putative morphological species; (**b**) Newly assigned *Dermogenys* species groups based on ABGD analysis.

**Figure 4:**
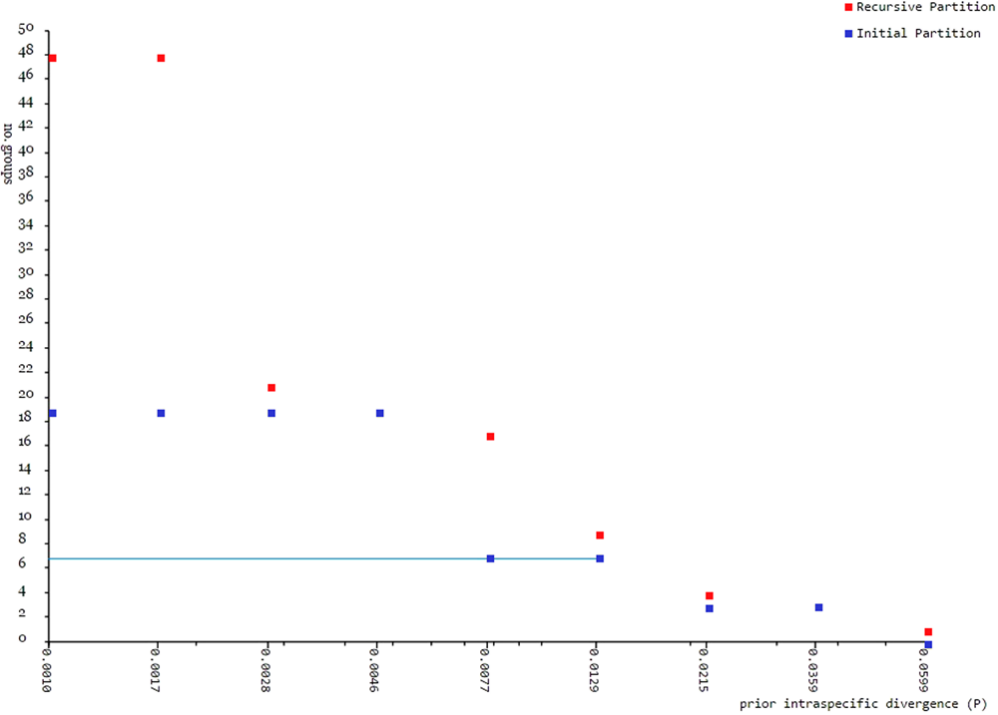
The number of genetically distinct OTUs according to the prior intraspecific divergence value generated by ABGD based on K2P distance. Data are from 299 molecular sequences.

A barcode gap analysis (Fig. 3(a)) incorporating current data and existing *D*. *pusilla* barcodes revealed that “barcode gaps” were present among all pairwise comparisons of the six putative *Dermogenys* species, indicating that all six groups were comprised of members belonging to different putative species. Following this, Automatic Barcode Gap Discovery (ABGD) analysis generated 3 to 48 OTUs (Fig. 4). However, an *a priori* intraspecific divergence of (*P*) (*P* = 0.0077–0.0129), chosen based on Fig. 4, generated 7 OTUs, instead of the expected six. The additional OTU (with respect to the six presumed species) identified by ABGD divided *D*. *bispina* into two groups, the first is from Kudat and the second from Sandakan (north and north-east Sabah, respectively).

The K2P genetic distances of the newly assigned groupings (based on OTUs) are summarised in Table 3. The minimum genetic distance values of the newly assigned grouping were slightly lower than for the six presumed *Dermogenys* species, with intraspecific distance ranging between 0.1% - 1.5% and interspecific distance from 2.7% to 13.2%. Average K2P genetic distance generated for the two groups of *D*. *bispina* (Kudat and Sandakan) is 2.7%. Overall, the p-distance within-site, intraspecific and intraspecific values were slightly lower than K2P value (Tables 2 and 3). A re-analysis of the barcode gap was conducted for the newly assigned groups, showing the presence of barcode gaps as presented in Fig. 3(b).

**Table 3:** Interspecific and intraspecific mean genetic distances based on K2P and p-distance (in parenthesis) among the newly assigned *Dermogenys* group after ABGD analysis.

	Species	1	2	3	4	5	6	7	8	9
1	*D*. *collettei*	0.011 (0.011)								
2	*D*. *sumatrana*	0.038 (0.037)	0.008 (0.008)							
3	*D*. *siamensis*	0.063 (0.060)	0.061 (0.058)	0.014 (0.013)						
4	*Dermogenys* sp.	0.092 (0.085)	0.098 (0.090)	0.112 (0.102)	0.002 (0.002)					
5	*D*. *bispina* Sandakan	0.126 (0.103)	0.112 (0.110)	0.121 (0.115)	0.123 (0.112)	0.002 (0.002)				
6	*D*. *bispina* Kudat	0.126 (0.099)	0.108 (0.108)	0.119 (0.114)	0.132 (0.119)	0.027 (0.027)	0.001 (0.001)			
7	*D*. *pusilla*	0.045 (0.044)	0.037 (0.036)	0.071 (0.067)	0.096 (0.089)	0.117 (0.108)	0.122 (0.112)	0.015 (0.015)		
8	*Nomorhamphus* sp.	0.164 (0.138)	0.169 (0.142)	0.171 (0.148)	0.150 (0.134)	0.151 (0.145)	0.156 (0.145)	0.173 (0.144)	n/c (n/c)	
9	*H*. *kuekenthali*	0.218 (0.188)	0.235 (0.202)	0.214 (0.188)	0.212 (0.185)	0.199 (0.170)	0.198 (0.174)	0.232 (0.199)	0.202 (0.179)	n/c (n/c)

n/c – no calculation due to single sample.

The COI gene tree estimated using ML and BI was congruent with that estimated using the NJ method (Fig. 2), with all tree estimation methods yielding results that recover two strongly supported geographic clusters of *D*. *bispina*, Kudat and Sandakan, reflecting groups identified in the ABGD analysis. At three locations (Kuala Sepetang (SPP), Sungai Iyu (SI) and Alur Itam(AI)), individuals were present from two taxonomic groups, namely *D*. *collettei* and *Dermogenys* sp.

## Discussion

The present study successfully identified and characterized six putative species of *Dermogenys*, including two reciprocally monophyletic groups in *D*. *bispina*; generated a reference barcode database for *Dermogenys*; and assessed levels of morphological/molecular disparity. Our preliminary *Dermogenys* taxonomic identification using morphological characters was limited, as the morphological keys^11^ are only applicable at particular life stages and are gender-specific. Therefore, due to a strong reliance on assessment of anal fin structure, which is only accurate through advanced radiographic analyses, conflict was common between morphologically identified specimens and results from our molecular analysis. These issues compound the mislabelling of *Dermogenys* species throughout museum collections and the taxonomic literature^11,20,57–59^. Failure to adequately discriminate to species level prior to biological and evolutionary investigations can also be problematic, for example de Bruyn *et al*.^12^ found major taxonomic ambiguities in their molecular analysis of *Dermogenys*, as morphologically recognised species were shown to comprise multiple, reciprocally monophyletic lineages. These taxonomic uncertainties are understandable, given the lack of easily distinguishable diagnostic characters for the group, yet ongoing mislabelling of specimens adds to taxonomic confusion and can lead to counter-productive conservation and management efforts. Thus, methods such as DNA barcoding may offer a means to ensure identifications are standardised, at least until such time as current taxonomic keys receive necessary revisions, alleviating the inconsistencies highlighted above, and the strong reliance on sex-specific characters.

The DNA barcoding approach relies, as to some extent do taxonomic keys, on expert knowledge to ascertain the identify of reference specimens, pulling together taxonomic literature and geographical information to compile a reference library. This paper represents the first attempt to do so for *Dermogenys*, verifying that the standard fish barcoding primers of Ward *et al*.^31^ work for barcoding the genus and adding data for five more *Dermogenys* species to the existing reference library of one. The DNA barcodes generated here enable the identification not only of adult specimens without undertaking complicated morphological investigations, but also of under-developed sub-adults, in the current example and more importantly, for future researchers seeking to identify *Dermogenys*.

The use of barcoding has already revealed some new information on geographical distributions of the genus. In clarifying the identity of fishes from Kuala Sepetang, Sg. Iyu and Alur Itam that were all tentatively identified as *D*. *collettei* using morphological and geographical information, our survey revealed that two genetically divergent species exist in sympatry at these sites, one of which remains undescribed. Such findings show that DNA barcoding could assess species diversity through the pairing of genetic distance methods and the genotypic cluster concept^60^. This study also lends further support that DNA barcoding may aid in larval identification, as has been observed in previous studies^36–38^, as well as in the identification of cryptic sympatric species, one of the known strengths of the barcoding approach^32,61^.

Of all taxa surveyed here, the *Dermogenys* species from northern Borneo exhibited among the highest intraspecific distance values, with some analyses indicating cause for considering the two geographically and genetically distinct populations, *D*. *bispina* ‘Kudat’ (north Borneo) and *D*. *bispina* ‘Sandakan’ (northeast Borneo), as sub-species. Borneo boasts a large number of endemic freshwater fishes^62,63^, and the island’s physical and environmental characteristics, as well as a paleo history of montane regions are believed to be core reasons for the north, in particular, being a centre for speciation and endemism^64,65^. Between Sandakan and Kudat in northern Borneo lies the Crocker Range (average height 1,800 m) separating eastern and western drainages^64,66^, and the potential cause for isolating the two *D*. *bispina* lineages, leading to formation of subspecies in allopatry. This range has previously been hypothesised as a barrier to gene flow for freshwater fishes in the region^67,68^.

Minimum distance between the two *D*. *bispina* groups was 2.7%, and this would be high enough to qualify as diagnostic of different species if applying a 2% divergence threshold (e.g., Ward *et al*.^30,69^. Hubert *et al*.^70^). However, the sole reliance of a 2% cut-off value for delimiting species across all taxa can mask the real diversity (e.g., Australian fishes^31^; Neotropical freshwater fishes^34^; Canadian freshwater fishes^70^; Tuna species^71^; North America’s freshwater fishes^72^) in the group as the initial intraspecific distance of the *D*. *bispina* group (when both Kudat and Sandakan were combined) was only 1.5%, and the initial barcode gap displayed no taxonomic ambiguity. This highlights the need for conducting comprehensive analyses as illustrated in the ABGD analysis. Combined with the lack of clear morphological differences, two OTUs supported with barcode gap re-analysis, the discovery of the two reciprocally monophyletic and geographically isolated *D*. *bispina* groups, probably warrants the two populations be considered at the very least as discrete evolutionary significant units or subspecies, and potentially as two different species, given that other reciprocally monophyletic groups with similar levels of divergence are classified as different species (e.g., *D*. *pusilla* and *D*. *sumatrana* - 3.7%). No overlap of intraspecific and interspecific genetic distance was observed. In fact, lower levels of nearest neighbour distance (NND) were observed in Canadian freshwater fishes^70^. Out of 190 barcoded species, 14 showed <0.1% NND value, 20 showed 0.1–1.0%, 17 showed 1.0–2.7%. A similar pattern was observed in Nigerian freshwater fishes^73^. O’Brien & Mayr^74^ outlined several criteria for subspecies classification 1) subspecies members share unique geographic range or habitat, 2) the OTUs are reciprocally monophyletic indicating that the genetic divergence of subspecies accumulated in the absence of gene flow, and is time-dependent and 3) unique natural history relative to other subdivisions of the species.

Even though DNA barcoding is a very effective tool for the systematics and validation of numerous freshwater fish taxa^32,34,70,75^, this approach leans heavily on the work of classical taxonomists, including the primary documentation of species and distributions, and ongoing work in validating and describing new OTUs. Morphological identification is likely to remain a fundamental approach for taxonomic identifications in most instances, and where DNA barcodes find no match in the barcode libraries, morphology remains the first port of call to validate a specimen’s identity^76^. Nevertheless, ambiguity in molecular findings is a good indication that knowledge of a taxonomic group is incomplete, and thus, DNA barcoding functions as a complementary and supporting tool for the robust identification of fish taxa and other organisms.

## Conclusion

This study reinforces the complementarity of both morphological and molecular characters as well as other lines of evidence (geographical distribution) in elucidating the taxonomic status and systematics of the *Dermogenys* group. The data presented contributes DNA reference barcodes for five additional species in this taxonomically challenging group, and we shed light on the level of genetic divergence expected within and between species in this genus, highlighting an area of Northern Borneo where geographically distinct lineages have arisen below the recognised species boundary. Taxonomy and systematics of the group remains incomplete, and detailed taxonomic work will be required to formally describe the new OTU (designated here as *Dermogenys* sp.), and to update taxonomic keys accordingly. Never-the-less, DNA barcoding as employed here demonstrates the power of molecular techniques in helping tackle difficult issues in taxonomy.

## Methods

### Collection of tissue samples

This study was carried out in accordance with the recommendations and approval by the Universiti Sains Malaysia Animal Ethics Committee. A total of 288 individuals from 31 locations were sampled from the inland waters of Malaysia, Western Indonesia, Thailand and Vietnam (Fig. 1, Table 1). Samples were obtained from slow moving brackish and freshwater systems, and collected using a scoop net with mesh size of 4 mm. The specimens were identified to genus level based on Rainboth^20^. Specimens were anesthetized with *Transmore* (NIKA Trading Co.), a fish stabilizer commonly used in aquatic trading prior to taking tissue samples from the pectoral fin (stored in 95% ethanol for DNA extraction). The specimens were then fixed with formalin and preserved in 70% ethanol. Back at the laboratory species level identification was conducted based on osteological characters of the modified anal fin (andropodium) in males^11^. The radiological study was conducted at the Chinese Academy of Sciences, Beijing. Preliminary identification was conducted based on morphological keys. As many characters are common in groups of species (e.g. melanophores from anterior to anal fin arranged into thin line observed in *D*. *collettei* and *D*. *siamensis* females; black pigment on distal tips of the posterior dorsal fin present in *D*. *collettei*, *D*. *siamensis*, *Dermogenys* sp. and *D*. *bispina* males; black pigment at the base and distal tip of pelvic fin observed in *D*. *collettei*, *D*. *sumatrana*, *D*. *siamensis*, and *Dermogenys* sp. males), precise identifications considered current knowledge on species distributions^1,11,16,77^. Thus, samples from Sabah, Vietnam, Thailand and Western Indonesia were putatively assigned to *D*. *bispina*, *D*. *siamensis* and *D*. *sumatrana* respectively, as a working hypothesis. Although de Bruyn *et al*.^12^ did not identify their specimens to species level, they hypothesised that most species were restricted in distribution range, which was further supported by their phylogeny, and therefore this classification scheme was justified in our study. In addition, two samples - morphologically identified as *D*. *collettei*, were contributed by the Florida Museum of Natural History (DC652 and DC683), making a total of 290 samples from 33 sampling sites (Fig. 1).

### Extraction, COI amplification and DNA sequencing

Total genomic DNA was extracted by using a modified conventional salt extraction procedure^78^. The template DNA was amplified by PCR in a 25 μL mixture containing 2.0 μL of DNA, 2.5 μL 10 × PCR buffer, 3.3 μL of 25 mM MgCl_2_, 0.5 μL 10 mM dNTPs, 0.25 μL 10 μM primers, 0.1 μL *i-Taq* plus polymerase and 16.1 μL DNAse-free water. The primers used for the amplification of the COI gene^31^ were Fish-F2 5′-TCG ACT AAT CAT AAA GAT ATC GGC AC-3′ and Fish-R2 5′-ACT TCA GGG TGA CCG AAG AAT CAG AA-3′. Amplifications were performed using a BioRad Thermocycler at 94 °C initial denaturation and 34 cycles with the following conditions: 20 s at 94 °C, 20 s at 47.9 °C and 70 s at 72 °C. The PCR products were sent for sequencing to 1st BASE Sequencing Service Sdn. Bhd. (Malaysia).

### Sequence analysis

Sequences were aligned using MEGA v6.06^79^ software, this package was also used to investigate base composition, and to calculate the number of variable sites and genetic distance measures. Both uncorrected pairwise distance (p-distance) and distance estimates based on the Kimura 2-parameter (K2P) model^80^ were calculated to assess mean “intrapopulation”, intraspecific and interspecific genetic distance within sample sites, and within and between species. Using the same software, phylogenetic relationships among haplotypes were constructed applying Neighbour-Joining (NJ) and Maximum Likelihood (ML) methods with 1000 bootstrap replicates. Tree construction was conducted using the K2P model^80^ for NJ analysis and Hasegawa-Kishino-Yano model^81^ with gamma (HKY + G) rates (optimal substitution model estimated by the model test run in MEGA v6.06) for ML analysis.

The relationships among haplotypes were also assessed using a Bayesian Inference (BI) method together with a Markov Chain Monte Carlo (MCMC) algorithm. In order to construct gene trees, PartitionFinder v1.1.0^82^ was used to determine the best-fit partitioning schemes and models of molecular evolution for phylogenetic analysis. The BI analyses were performed using MrBayes^83^ with employment of HKY + I^81^, F81^84^, and GTR + G^85^ for the first, second and third codon, 1 million MCMC chains and a 50% burn in. The trace files generated from MrBayes run were diagnosed in Tracer v1.6^86^ to evaluate the MCMC chain. The COI trees generated were visualized and edited using FigTree v1.4.2^87^. *Nomorhamphus* sp. (GenBank Acc. No JQ430374) and *Hemirhamphodon* (Acc. No KM405787) were included as outgroups^88^. Additional *D*. *pusilla* (Acc. KU692464 to KU692472) COI sequences from GenBank were included to improve phylogenetic resolution.

The maximum intraspecific distance against the minimum nearest-neighbour distance graph was plotted to check the presence of a “barcode gap” in the dataset^89^. Presence of barcode gaps among sets of sequences within a presumed species/taxon indicates that there is likely to be more than a single taxon within the group. The number of operational taxonomic units (OTUs) based on pairwise sequence distances between individuals within the dataset was generated using the Automatic Barcode Gap Discovery (ABGD)^90^ species delineation tool on a web interface (http://wwwabi.snv.jussieu.fr/public/abgd/abgdweb.html) with default settings and the K2P model employed. The interpretation of the ABGD results is very straightforward. An OTU is considered as successfully delimited when the predicted groups are formed and no other unrelated sequences were included in that group^91^.

### Data availability

The datasets generated during and/or analysed during the current study are available from the corresponding author on reasonable request.
